# Advancing Soybean Improvement: Multi-Omics Strategies, Cutting-Edge Techniques, and Bioinformatics Innovations

**DOI:** 10.3390/plants15101533

**Published:** 2026-05-18

**Authors:** Bahram Samanfar

**Affiliations:** Agriculture and Agri-Food Canada, Ottawa Research and Development Centre (AAFC-Ottawa RDC), Ottawa, ON K1A 0C6, Canada; bahram.samanfar@agr.gc.ca

Plant science research has entered a transformative era driven by rapid technological developments in high-throughput sequencing, omics-based approaches, computational biology, and precision phenotyping. These advances are enabling researchers to dissect complex biological processes at an unprecedented resolution and scale. As global agriculture faces mounting pressures—including climate change, emerging pathogens, soil degradation, and increasing food demand—the ability to translate fundamental discoveries into practical crop improvement strategies has become more important than ever. Journals such as *Plants* play an essential role in disseminating advances across multiple disciplines of plant biology, including genetics, physiology, ecology, and biotechnology.

This Special Issue, entitled “Advancing Soybean Improvement: Multi-Omics Strategies, Cutting-Edge Techniques, and Bioinformatics Innovations”, brings together a diverse collection of research articles, methodological developments, and comprehensive reviews that address contemporary challenges in soybean research. Soybean (*Glycine max* L.) is one of the most important crops worldwide, serving as a primary source of plant-derived protein and oil for food, feed, and industrial applications. Improving soybean productivity, resilience, and nutritional value is therefore a central goal of modern agricultural research.

The studies assembled in this Special Issue highlight recent advances in plant genetics, molecular biology, physiology, and biotechnology, with a strong emphasis on understanding plant responses to environmental stresses and improving crop productivity and resilience. By integrating approaches ranging from functional genomics to systems biology, multi-omics integration, and advanced breeding strategies, the contributions provide valuable insights into the mechanisms that govern plant growth, adaptation, and yield formation.

Importantly, this Special Issue also emphasizes the growing role of integrative computational biology and multi-omics frameworks in accelerating soybean improvement. Haidar et al. [1] provide a comprehensive review of emerging multi-omics strategies and bioinformatics innovations in soybean research, highlighting how the integration of genomics, transcriptomics, proteomics, metabolomics, phenomics, and artificial intelligence-driven analytics is reshaping modern breeding pipelines. Their review underscores the importance of systems-level approaches to identifying key regulatory networks, improving predictive breeding, and enhancing resilience to environmental stresses.

Collectively, the articles included in this Special Issue address several key themes that are central to contemporary plant science research. These include the genetic and molecular basis of agronomic traits, plant responses to biotic and abiotic stresses, advances in functional genomics and transcriptomics, emerging strategies for sustainable crop production, and increased integration of machine learning and bioinformatics tools into crop improvement programs.

**Genetic Architecture of Agronomic Traits**

Understanding the genetic basis of complex traits remains a cornerstone of modern plant breeding. Several articles in this Special Issue explore the genetic architecture underlying important agronomic characteristics, including yield components, seed composition, and stress tolerance. Through the integration of genome-wide association studies, quantitative trait locus (QTL) mapping, and comparative genomic analyses, these studies identify candidate genes and regulatory pathways that contribute to phenotypic variation in soybean.

For example, Potapova et al. [2] conducted a genome-wide association study using imputed genotypes across a diverse panel of Eurasian soybean varieties to identify loci associated with seed oil and protein content. Their findings contribute to a better understanding of the genetic determinants underlying seed composition traits that are critical for soybean nutritional and industrial value.

Similarly, Yoosefzadeh-Najafabadi et al. [3] employed support vector regression-mediated GWAS to identify durable genomic regions associated with soybean seed quality traits. By integrating machine learning with association mapping, this study demonstrates how advanced analytical methods can enhance the detection of complex genetic signals controlling economically important traits.

In another contribution, Ćeran et al. [4] investigated strategies to optimize genomic prediction models through selective genotyping and phenotyping across populations with different levels of genetic diversity. Their work highlights the importance of population structure and sampling design in improving the accuracy and efficiency of genomic selection approaches in soybean breeding programs.

**Functional Genomics and Molecular Regulation**

Another major theme that emerges in this Special Issue is the application of functional genomics to unravel the regulatory mechanisms controlling plant development and stress responses. Advances in transcriptomics, gene expression profiling, and network analysis have made it possible to identify key regulatory genes and molecular modules that orchestrate complex biological processes.

Zhang et al. [5] performed a genome-wide identification and characterization of the fatty acid export (FAX) gene family in soybean and revealed the functional importance of *GmFAX8* in regulating fatty acid transport and oil accumulation in seeds. Their results provide new insights into lipid metabolism and identify promising molecular targets for enhancing soybean oil content.

Li et al. [6] demonstrated that knockout of the cytokinin oxidase gene *GmCKX3* enhances soybean seed yield through cytokinin-mediated cell expansion and increased lipid accumulation. This study highlights the critical role of hormonal regulation in controlling seed development and provides an example of how targeted gene manipulation can improve yield-related traits.

Another notable study by Zhao et al. [7] investigated the soybean *AlkB* homolog gene family and demonstrated that *GmALKBH10B* genes function as RNA m6A demethylases involved in post-transcriptional regulation. The authors further explored the expression patterns of these genes under abiotic stress conditions, providing new insights into epigenetic regulation in soybean.

**Integration of Multi-Omics Approaches**

Multi-omics integration represents one of the most powerful approaches to dissecting complex biological traits. By combining genomic, transcriptomic, metabolomic, and phenotypic datasets, researchers can uncover regulatory networks that link molecular variation with observable plant traits.

Liang et al. [8] conducted an integrated transcriptomic and metabolomic analysis to investigate anthocyanin accumulation in black soybean seed coats under low-nitrogen conditions. Their findings reveal how nitrogen availability influences metabolic pathways and transcriptional regulation associated with anthocyanin biosynthesis, highlighting the complex interplay between nutrient status and secondary metabolism.

Razgonova et al. [9] combined confocal laser microscopy with high-resolution mass spectrometry to analyze autofluorescence signatures and metabolite profiles across multiple soybean varieties. This innovative metabotyping approach provides a detailed characterization of bioactive compounds and demonstrates the potential of advanced imaging and metabolomic technologies for exploring biochemical diversity in the soybean germplasm.

The review by Haidar et al. [1] further complements these studies by outlining how integrated multi-omics platforms, coupled with bioinformatics pipelines and predictive modeling approaches, are enabling a systems-level understanding of soybean biology. The authors emphasize that combining omics datasets with machine learning frameworks can substantially improve trait prediction, candidate gene prioritization, and breeding efficiency.

**Biotic and Abiotic Stress Responses**

Abiotic stresses such as drought and salinity represent major constraints on crop productivity worldwide. With climate change intensifying the frequency and severity of extreme environmental conditions, improving crop resilience has become a key priority in agricultural research.

Sun et al. [10] investigated the genetic basis of salt tolerance at the soybean seedling stage using both genome-wide association analysis and linkage mapping. Their study identified several QTLs associated with salt tolerance and proposed candidate genes involved in stress response pathways, providing valuable genetic resources for developing salt-tolerant soybean cultivars.

Shekoofa et al. [11] examined the inheritance of early stomatal closure in a soybean mapping population under drought conditions. Early stomatal closure is an important physiological mechanism that helps plants conserve water during drought stress. By characterizing the genetic basis of this trait, the study contributes to breeding strategies aimed at improving drought resilience.

Biotic stress resistance is addressed by Liu et al. [12], who performed fine mapping of quantitative trait loci associated with resistance to Race 7 of the fungal pathogen *Cercospora sojina*, the causal agent of frogeye leaf spot disease. By combining linkage analysis and genome-wide association studies, the authors identified candidate resistance genes that could facilitate the development of disease-resistant soybean varieties.

Also exploring disease resistance, Maquil et al. [13] investigated the genetic basis of partial resistance to soybean rust under field conditions using FarmCPU and machine learning approaches. Their study identified genomic regions associated with rust resistance and demonstrated the effectiveness of integrating advanced statistical and machine learning frameworks for detecting complex resistance-associated loci. This work further highlights the growing importance of artificial intelligence-assisted approaches in soybean disease resistance breeding.

**Nutritional and Functional Compounds**

Beyond yield and stress tolerance, soybean is also valued for its nutritional and bioactive compounds. Locklear et al. [14] investigated the genetic basis of lunasin accumulation, a peptide with reported anticancer and antioxidant properties. Through multi-locus genome-wide association analysis, the authors identified quantitative trait nucleotides and candidate genes associated with lunasin content, offering new opportunities to enhance the health-promoting properties of soybean-derived foods.

**Technological Innovations for Genomic Research**

Advances in biotechnology and automation are increasingly supporting large-scale genomic studies and breeding programs. In this Special Issue, Boucher St-Amour et al. [15] introduce a semi-automated robotic DNA extraction protocol known as RoboCTAB. This high-throughput method enables efficient and consistent DNA extraction for large-scale genotyping projects, addressing a major bottleneck in genomic research and breeding pipelines.

**Emerging Trends and Future Perspectives**

Taken together, the studies published in this Special Issue highlight several important trends in modern plant science research. Firstly, the integration of multi-omics datasets is enabling researchers to explore plant biology at a systems level, linking genetic variation with metabolic and physiological responses. Secondly, advanced computational methods, including machine learning, artificial intelligence, and genomic prediction models, are enhancing the ability to detect complex genetic signals associated with agronomic traits. Thirdly, innovations in experimental methodologies and automation are accelerating data generation and analysis, thereby facilitating large-scale genomic studies.

The inclusion of studies such as those by Haidar et al. [1] and Maquil et al. [13] further demonstrates how computational biology, machine learning, and integrated omics platforms are increasingly converging to drive next-generation soybean improvement strategies. These interdisciplinary approaches are expected to play a critical role in developing climate-resilient, high-yielding, and nutritionally enhanced soybean cultivars.

Looking ahead, continued progress in soybean improvement will depend on interdisciplinary collaboration among geneticists, physiologists, molecular biologists, bioinformaticians, and breeders. The integration of emerging technologies such as artificial intelligence, high-throughput phenotyping, and genome editing will further enhance our capacity to understand complex plant traits and develop improved cultivars.

**Concluding Remarks**

The Guest Editors would like to express their sincere gratitude to all authors who contributed their high-quality research to this Special Issue, as well as to the reviewers whose constructive feedback helped ensure the scientific rigor of the published articles. We also thank the editorial team of *Plants* for their invaluable support throughout the editorial and publication process.

The research presented in this Special Issue demonstrates the breadth and dynamism of contemporary soybean science. By advancing our understanding of plant genetics, physiology, metabolism, stress adaptation, and computational biology, these studies provide important foundations for developing innovative strategies for crop improvement and sustainable agriculture.

We hope that the insights presented in this Special Issue will stimulate further research, foster interdisciplinary collaborations, and contribute to the continued advancement of soybean biology and crop science.

## References

[1] Haidar S., Hooker J., Lackey S., Elian M., Puchacz N., Szczyglowski K., Marsolais F., Golshani A., Cober E.R., Samanfar B. (2024). Harnessing Multi-Omics Strategies and Bioinformatics Innovations for Advancing Soybean Improvement: A Comprehensive Review. Plants.

[2] Potapova N.A., Zorkoltseva I.V., Zlobin A.S., Shcherban A.B., Fedyaeva A.V., Salina E.A., Svishcheva G.R., Aksenovich T.I., Tsepilov Y.A. (2025). Genome-Wide Association Study on Imputed Genotypes of 180 Eurasian Soybean *Glycine max* Varieties for Oil and Protein Contents in Seeds. Plants.

[3] Yoosefzadeh-Najafabadi M., Torabi S., Tulpan D., Rajcan I., Eskandari M. (2023). Application of SVR-Mediated GWAS for Identification of Durable Genetic Regions Associated with Soybean Seed Quality Traits. Plants.

[4] Ćeran M., Đorđević V., Miladinović J., Vasiljević M., Đukić V., Ranđelović P., Jaćimović S. (2024). Selective Genotyping and Phenotyping for Optimization of Genomic Prediction Models for Populations with Different Diversity. Plants.

[5] Zhang Y., Zhu Y., Rui X., Li Y., Wang J., Zhan Y., Li Y., Zhao X., Han Y., Zhao X. (2025). Genome-Wide Identification and Analysis of the Fatty Acid Export Family Revealed the Role of *GmFAX8* in Improving Soybean Oil Accumulation. Plants.

[6] Li X., Qian X., Zhao F., Niu L., Zhang Y., Han S., Hao D., Chen Z. (2025). Knockout of *GmCKX3* Enhances Soybean Seed Yield via Cytokinin-Mediated Cell Expansion and Lipid Accumulation. Plants.

[7] Zhao J., Yang T., Liu P., Liu H., Zhang H., Guo S., Liu X., Chen X., Chen M. (2024). Genome-Wide Identification of the Soybean *AlkB* Homologue Gene Family and Functional Characterization of GmALKBH10Bs as RNA m^6^A Demethylases and Expression Patterns under Abiotic Stress. Plants.

[8] Liang S., Si F., Guo C., Chai Y., Yang T., Wang P. (2025). Integrated Analysis of Transcriptome and Metabolome Reveals the Accumulation of Anthocyanins in Black Soybean (*Glycine max* L.) Seed Coats Induced by Low Nitrogen Concentration in the Nutrient Solution. Plants.

[9] Razgonova M.P., Navaz M.A., Butovets E.S., Lukyanchuk L.M., Chunikhina O.A., Ercişli S., Emelyanov A.N., Golokhvast K.S. (2025). Autofluorescence and Metabotyping of Soybean Varieties Using Confocal Laser Microscopy and High-Resolution Mass Spectrometric Approaches. Plants.

[10] Sun M., Zhao T., Liu S., Han J., Wang Y., Zhao X., Li Y., Teng W., Zhan Y., Han Y. (2024). QTL Detection of Salt Tolerance at Soybean Seedling Stage Based on Genome-Wide Association Analysis and Linkage Analysis. Plants.

[11] Shekoofa A., Moser V., Dhakal K., Poudel I., Pantalone V. (2023). Inheritance of Early Stomatal Closure Trait in Soybean: Ellis × N09-13890 Population. Plants.

[12] Liu Y., Hu B., Yu A., Liu Y., Xu P., Wang Y., Ding J., Zhang S., Li W.-X., Ning H. (2025). Fine Mapping of QTLs/QTNs and Mining of Genes Associated with Race 7 of the Soybean *Cercospora sojina* by Combining Linkages and GWAS. Plants.

[13] Maquil A.D.P., Obua T., Nsibo D.L., Ochwo-Ssemakula M., Murithi H., Gibson P., Garcia-Oliveira A.L., Edema R., Dramadri I., Yoosefzadeh-Najafabadi M. (2026). Identification of Genomic Regions for Partial Resistance to Soybean Rust Under Field Conditions Using FarmCPU and Machine Learning Approaches. Plants.

[14] Locklear R., Kusumah J., Rashad L., Lugaro F., Viera S., Kipyego N., Kipkosgei F., Jerop D., Jacquet S., Kassem M.A. (2025). Multi-Locus GWAS Mapping and Candidate Gene Analysis of Anticancer Peptide Lunasin in Soybean (*Glycine max* L. Merr.). Plants.

[15] Boucher St-Amour V.-T., Tomar V., Belzile F. (2025). High-Throughput DNA Extraction Using Robotic Automation (RoboCTAB) for Large-Scale Genotyping. Plants.

